# Ansofaxine hydrochloride inhibits tumor growth and enhances Anti-TNFR2 in murine colon cancer model

**DOI:** 10.3389/fphar.2023.1286061

**Published:** 2023-12-14

**Authors:** Qianyu Jing, Quan Wan, Yujie Nie, Junqian Luo, Xiangyan Zhang, Lan Zhu, Huan Gui, Linzhao Li, Chenglv Wang, Shuanghui Chen, Mengjiao Wang, Haohua Yuan, Hang Lv, Runsang Pan, Qianjun Jing, Yingjie Nie

**Affiliations:** ^1^ School of Basic Medical Sciences, Zunyi Medical University, Zunyi, China; ^2^ NHC Key Laboratory of Pulmonary Immunological Diseases, Guizhou Provincial People’s Hospital, Guiyang, China; ^3^ The First People’s Hospital of Jinzhong, Jinzhong, China; ^4^ School of Medicine, Guizhou University, Guiyang, China; ^5^ Guizhou Medical University, Guiyang, China; ^6^ Chongqing Medical University, Chongqing, China

**Keywords:** ansofaxine hydrochloride, anti-TNFR2, exhausted CD8 + T cells, Tregs, tumor immunotherapy, colon cancer

## Abstract

**Introduction:** As psychoneuroimmunology flourishes, there is compelling evidence that depression suppresses the anti-tumor immune response, promotes the progression of cancer, and inhibits the effectiveness of cancer immunotherapy. Recent studies have reported that antidepressants can not only alleviate the depressant condition of cancer patients, but also strengthen the anti-tumor immunity, thus suppressing tumors. Tumor necrosis factor receptor 2 (TNFR2) antagonistic antibodies (Anti-TNFR2) targeting tumor-infiltrating regulatory T cells (Tregs) has achieved great results in preclinical studies, and with a favorable toxicity profile than existing immunotherapies, and is expected to become a new generation of more effective treatment strategies. Understanding the effects of combination therapy with antidepressants and Anti-TNFR2 may help design new strategies for cancer immunotherapy.

**Methods:** We treated CT26, HCT116, MCA38 and SW620 colon cancer cells with fluoxetine (0–50 µM), ansofaxine hydrochloride (0–50 µM) and amitifadine hydrochloride (0–150 µM) to examine their effects on cell proliferation and apoptosis. We explored the antitumor effects of ansofaxine hydrochloride in combination with or without Anti-TNFR in subcutaneously transplanted CT26 cells in tumor-bearing mouse model. Antitumor effects were evaluated by tumor volume. NK cell, M1 macrophage cell, CD4^+^ T cell, CD8^+^ T cell, exhausted CD8^+^ T and regulatory T cell (Tregs) subtypes were measured by flow cytometry. 5-hydroxytryptamine, dopamine and norepinephrine levels were measured by ELISA.

**Results:** Oral antidepression, ansofaxine hydrochloride, enhanced peripheral dopamine levels, promoted CD8^+^T cell proliferation, promoted intratumoral infiltration of M1 and NK cells, decreased the proportion of tumor-infiltrating exhausted CD8^+^T cells, and strengthened anti-tumor immunity, thereby inhibiting colon cancer growth. In combination therapy, oral administration of ansofaxine hydrochloride enhanced the efficacy of Anti-TNFR2, and produced long-term tumor control in with syngeneic colorectal tumor-bearing mice, which was attributable to the reduction in tumor-infiltrating Treg quantity and the recovery of CD8^+^ T cells function.

**Discussion:** In summary, our data reveal the role of ansofaxine hydrochloride in modulating the anti-tumor immunity. Our results support that exhausted CD8^+^T is an important potential mechanism by which ansofaxine hydrochloride activates anti-tumor immunity and enhances anti-tumor effects of anti-TNFR2.

## 1 Introduction

Colon cancer (CRC) is the fourth deadliest cancer worldwide, killing hundreds of thousands of people each year (Sung et al., 2021). At the same time, the mental health of patients is also attracting more and more attention, with the occurrence of depression in cancer patients being higher than any other disease patients (Yan et al., 2019; Yang et al., 2021a; Endo et al., 2022). There are convincing evidences that Depression promotes the growth of various cancers by regulating the neuroimmune system, and inhibits the efficacy of cancer treatment (Liu et al., 2015; Sommershof et al., 2017; Zhang et al., 2020). Therefore, research into therapies that can treat both cancer and depression at the same time is clinically valuable.

Currently, colon cancer treatment includes surgery, radiation, chemotherapy, targeted therapy, and immunotherapy. In recent years, immune checkpoint inhibitors such as anti-programmed cell death protein 1 (PD-1) and anti-cytotoxic T lymphocyte–associated protein 4 (CTLA-4) have achieved great results in cancer treatment, however, because to low response rates and immune-related adverse events (irAEs), most patients are unable to benefit (Fritz and Lenardo, 2019). Tumor necrosis factor receptor 2 (TNFR2), one of the two receptors that mediate the biological function of TNF, is involved in cancer cell growth (Vanamee and Faustman, 2017; Torrey et al., 2019; Li et al., 2023), and is high expressed in exhausted CD8T cells and tumor-infiltrating regulatory T cells (Tregs) in a variety of cancers (Liao et al., 2023). Now, there are convincing evidences that TNFR2 can serve as a key mediator involved in the activation and proliferation of Tregs, enhancing immunosuppressive function (Chen et al., 2007; Kawano et al., 2022). In addition, Other types of immunosuppressive cells, such as MDSCs, and some tumor cells also express TNFR2 (Vanamee and Faustman, 2017). Knocking down TNFR2 on cancer cells by CRISPR/Cas9 technology significantly impaired the growth of colon cancer, and increased the number of tumor-infiltrating IFN-γ^+^ CD8 cells (Li et al., 2023). TNFR2 antagonistic antibody (anti-TNFR2) inhibit Tregs by targeting TNFR2 *in vitro*, and kill TNFR2-expressing tumor cells and Tregs in advanced Sézary syndrome (Torrey et al., 2019; Moatti et al., 2022). Moreover, in mouse models, anti-TNFR2 was less toxic than anti-CTLA-4 (Tam et al., 2019). Therefore, Anti-TNFR2 is considered a very promising new tumor immunotherapy (Vanamee and Faustman, 2017). Currently, preclinical studies of human anti-TNFR2 antibodies have yielded very encouraging results, justifying their clinical development (Tam et al., 2019).

The combination of antidepressant and immune checkpoint inhibitor has also been reported. Fluoxetine and monoamine oxidase inhibitor (MAOI) were common clinical medications for depression, recent studies have shown that they not only suppress the adverse impact of depression, but also enhance the anti-tumor immunity of the body (Di Rosso et al., 2016; Di Rosso et al., 2018; Marcinkute et al., 2019; Wang et al., 2021a; Yang et al., 2021b; Schneider et al., 2021). Fluoxetine combined with anti-PD1 provides long-term tumor control in mouse models (Schneider et al., 2021). MAOI inhibits macrophage immunosuppressive polarization, and synergistically suppresses tumors in combination with anti-PD-1 therapy (Wang et al., 2021b). However, the effect of fluoxetine is slow and long-term use often induces adverse effects such as loss of appetite or sexual dysfunction, and MAOI was associated with an increased incidence of colorectal cancer (Lee et al., 2017). Triple reuptake inhibitors (TRIs) are believed to enhance neurotransmission in all monoamine systems, and have the advantage of acting quickly, and improving symptoms of sexual disorders and disorientation (Tran et al., 2012; Shao et al., 2014; Sharma et al., 2015). Ansofaxine hydrochloride (LY03005) is a new TRI for the treatment of major depressive disorder in adults, which has completed phase III clinical trials and is well tolerated (Mi et al., 2022). However, the role of ansofaxine hydrochloride in cancer immunotherapy is unclear. Therefore, we took the ansofaxine hydrochloride as a breakthrough point to study its effect on colon cancer.

Here, we found that ansofaxine hydrochloride, like fluoxetine, inhibits colon cancer cell growth *in vitro* by inducing apoptosis. In CT26 colon cancer model, ansofaxine hydrochloride enhanced the proportion of CD8^+^T cells in spleen, decreased the proportion of tumor infiltration exhausted CD8^+^T cells, and increased the proportion of natural killer cells (NKs) and M1 macrophages in spleen and tumor, which may be due to the enhancement of peripheral dopamine (DA) and the reduction of peripheral 5-hydroxytryptamine (5-HT), ultimately inhibited tumor growth. In combination therapy, ansofaxine hydrochloride enhanced the efficacy of anti-TNFR2 in colon cancer, enabling eradication of established tumors in 20% of mice, and triggering syngeneic tumor-specific systemic immunity. These results provide a new way of treating colon cancer patients with depression.

## 2 Methods and materials

### 2.1 Mice and cell culture

Female wild-type Balb/c mice (6- to 8-week-old) were obtained from Spaefer Biotechnology Co., Ltd. (Beijing, China) (Animal Quality Certificate: SCXK (Beijing) 2019-0008). Mice were housed in a specific pathogen-free (SPF) lab at the Experimental Animal Center of Guizhou University of Traditional Chinese (Animal experiment license: SYXK 2021-0005), and the experiments were started after 7 days of acclimatization to the surrounding environment. This animal experiment was approved by Ethics Committee of Guizhou Provincial People’s Hospital, China. The mouse colon cancer cell lines (CT26, MCA38) and mouse breast cancer cell line (4T1) were supplied by Pricella (Wuhan, China). The human colon cancer cell lines (HCT116, SW620) were gifted by Prof. Jie Ding (Guizhou Province People‘s Hospital, Guizhou, China). The cells were cultured in RPMI-1640 complete medium containing 10% fetal bovine serum (FBS), and supplemented with penicillin (100 units/mL), streptomycin (100 μg/mL) glutamine (2 mM), at 37°C under 5% CO2.

### 2.2 Chemicals

All chemicals were of analytical grade. Fluoxetine was purchased by Absin (Shanghai, China). Amitifadine hydrochloride (DOV 21947), ansofaxine hydrochloride (LY03005), cell counting kit-8 (CCK-8), and telratolimod (3M-052) were purchased by MedChenExpress (Monmouth Junction, NJ, United States). Anti-mouse TNFR2 (CD120b, clone TR75-54.7) was purchased by BioXCell (W. Lebanon, NH). High mobility group nucleosome binding protein 1 (HMGN1) was purchased by Bio-techne (R&D Systems. United States). ST/5-HT (Serotonin/5-hydroxytryptamine), DA (dopamine), and NA/NE (Noradrenaline/norepinephrine) ELISA Kit, and Annexin-V-FITC/PI were purchased by Elabscience Biotechnology Co., Ltd. (Wuhan, China). Fetal bovine serum (FBS) was purchased by Pricella (Wuhan, China). Penicillin-streptomycin stock solutions was purchased by New Cell and Molecular Biotech Co., Ltd. (Suzhou, China). Trypsin-EDTA (0.25%), EDTA- and phenol red-free trypsin (0.25%) were purchased by Servicebio (Wuhan, China). RPMI-1640 medium was purchased by GIBCO BRL (Grand Island, NY, United States). FITC-anti-mouse CD4 (clone GK1.5) was purchased by Biolegend (California, United States). BV510-anti-mouse CD45 (clone 30-F11), BV605-anti-mouse CD3 (clone 17A2), BV421-anti-mouse CD8 (clone 53-6.7), PerCPcy5.5-anti-mouse CD279 (clone J43), PE-anti-mouse CD223 (clone C9B7W), BV421-anti-mouse CD49b (clone DX5), FITC-anti-mouse CD11b (clone M1/70) and BV650-anti-mouse CD86 (clone GL1) were purchased by BD Biosciences (Franklin Lake, New Jersey, United States). PE-anti-mouse F4/80 (clone BM8) was purchased by eBioscience (California, United States).

### 2.3 Cell counting Kit-8 (CCK-8) assay

The cells (5,000 cells/well) were inoculated in the 96-well plates. After 24 h of incubation, the cells were treated with fluoxetine, amitifadine hydrochloride or ansofaxine hydrochloride for 24 h. The medium was then replaced to FBS-free RPMI-1640 with 10% CCK8 solution in a dark room. The OD values were collected at 450 nm after incubation at 37°C for 1 h.

### 2.4 Cell apoptosis analysis

Cells (2 × 10^5^ cells/well) were inoculated into 6-well plates. After 24 h of incubation, the cells were treated with amitifadine hydrochloride or ansofaxine hydrochloride at the appropriate concentration. After 24 h, all cells were collected and then incubated with PI-FITC antibody for 15 min. Detection was performed using a flow cytometer (BD FACSCelestaTM).

### 2.5 Construction and treatment of mouse model of CT26 colon cancer

CT26 colon cancer cells (2 × 10^5^/100 ul/mouse) were injected subcutaneously into the right abdomen of the subject mice. In rechallenge experiments, tumor-free mice surviving for 80 days were inoculated with CT26 cells (2 × 10^5^) in the left abdomen and 4T1 breast cancer cells (2 × 10^5^) in the right abdomen. “Survival” represents the time it takes for the tumor to progress to 2,000 mm^3^, which is the humane endpoint for triggering euthanasia. The tumor size was monitored every 3 days, and was calculated as: tumor size = (length × width^2^)/2.

Treatment was started when tumor volume reached 100 mm^3^. Ansofaxine hydrochloride was administered by gavage for 5 or 12 days at 300 μg in 0.1 mL of water. Tumors and spleens were taken from mice the day after the last treatment and studied. In combination treatment, ansofaxine hydrochloride (300 ug/100 ul) was administered by gavage, anti-TNFR2 (200 ug/200 ul) was injected intraperitoneally at days 2 and 7, HMGN1 (0.5 ug/50 ul) and 3M-052 (20 ug/50 ul) was injected intratumoral at days 1, 5 and 9.

### 2.6 Flow cytometry analysis

To analyze the proportions of CD8^+^T and exhausted CD8^+^T cells, cells were stained with BV510-CD45, BV605-CD3, BV421-CD8, PerCPcy5.5-CD279 and PE-CD223 antibodies. To analyze the proportions of CD4^+^T, Tregs and NK cells, cells were stained with BV510-CD45, BV605-CD3, FITC-CD4, BV421-CD49b and PE-Foxp3 antibodies. To analyze the proportions of M1, cells were stained with BV510-CD45, FITC-CD11b, PE-F4/80, and BV650-CD86 antibodies. All cells were assayed with flow cytometry and data analyzed with Flowjo 10 software.

### 2.7 Detection of neurotransmitters (5-HT, DA and NA/NE)

Cells (2 × 105 cells/well) are inoculated into 6-well plates. After 24 h of incubation, the cells were treated with 40 uM ansofaxine hydrochloride or solvent control. After 48 h, the cell supernatant was collected and then assayed by using ELISA. Mice were executed after ansofaxine hydrochloride treatment for 5 days, peripheral blood was collected, centrifuged at 3,000 rpm for 5 min, and the neurotransmitter levels of serum were measured by ELISA.

### 2.8 Statistical analysis

Two-tailed Student’s *t*-test, one-way ANOVA and Log-rank test were used to compare statistical differences between groups. *p*-value < 0.05 was statistically significant.

## 3 Results

### 3.1 Comparison of fluoxetine, ansofaxine hydrochloride and amitifadine hydrochloride for inhibitory effect in various colon cancer cell lines

**FIGURE 1:**
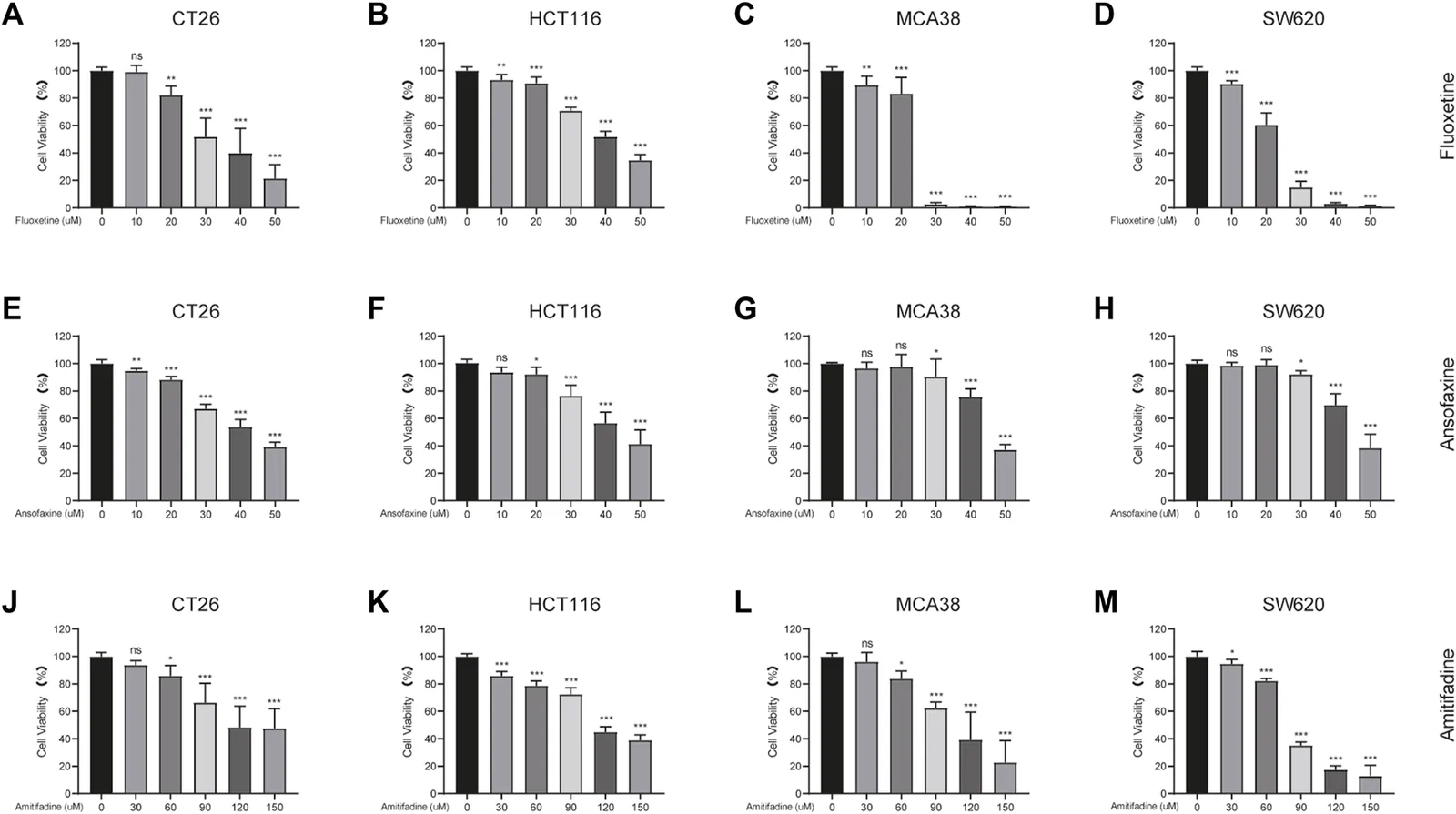
Comparison of fluoxetine, ansofaxine hydrochloride and amitifadine hydrochloride for inhibitory effect in various colon cancer cell lines. **(A–D)** CT26, MCA38, HCT116 and SW620 were treated for 24 h with fluoxetine (0, 10, 20, 30, 40, and 50 μM). **(E–H)** CT26, MCA38, HCT116, SW620 were treated for 24 h with Ansofaxine hydrochloride (0, 10, 20, 30, 40, and 50 μM). **(J–M)** CT26, MCA38, HCT116, SW620 cells were treated for 24 h with Amitifadine hydrochloride (0, 30, 60, 90, 120, and 150 μM). CCK8 for measurement of cell viability. Data are expressed as mean ± SD; *p < 0.05, **p < 0.01, ***p < 0.001.

Here, we chose fluoxetine and two TRIs, ansofaxine hydrochloride and amitifadine hydrochloride, as subjects of study. The inhibitory effect of fluoxetine, ansofaxine hydrochloride and amitifadine hydrochloride on different types of colon cancer cells were assayed by CCK8. For human colon cancer cell lines (HCT116, SW620), the dates suggested that ansofaxine hydrochloride and amitifadine hydrochloride inhibited the cell proliferation in a dose-dependent manner such as fluoxetine (Figures 1B, D, F, H, K, M). We then compared the inhibitory effects of the drugs on mouse colon cancer cell lines (CT26, MCA38). The data showed that all drugs inhibited the growth of CT26 and MCA38 (Figures 1A, C, E, G, J, L). Therefore, we thought that two TRIs have the antitumor activity *in vitro* such as fluoxetine.

### 3.2 TRIs induced apoptosis of multiple colon cancer cell lines

**FIGURE 2:**
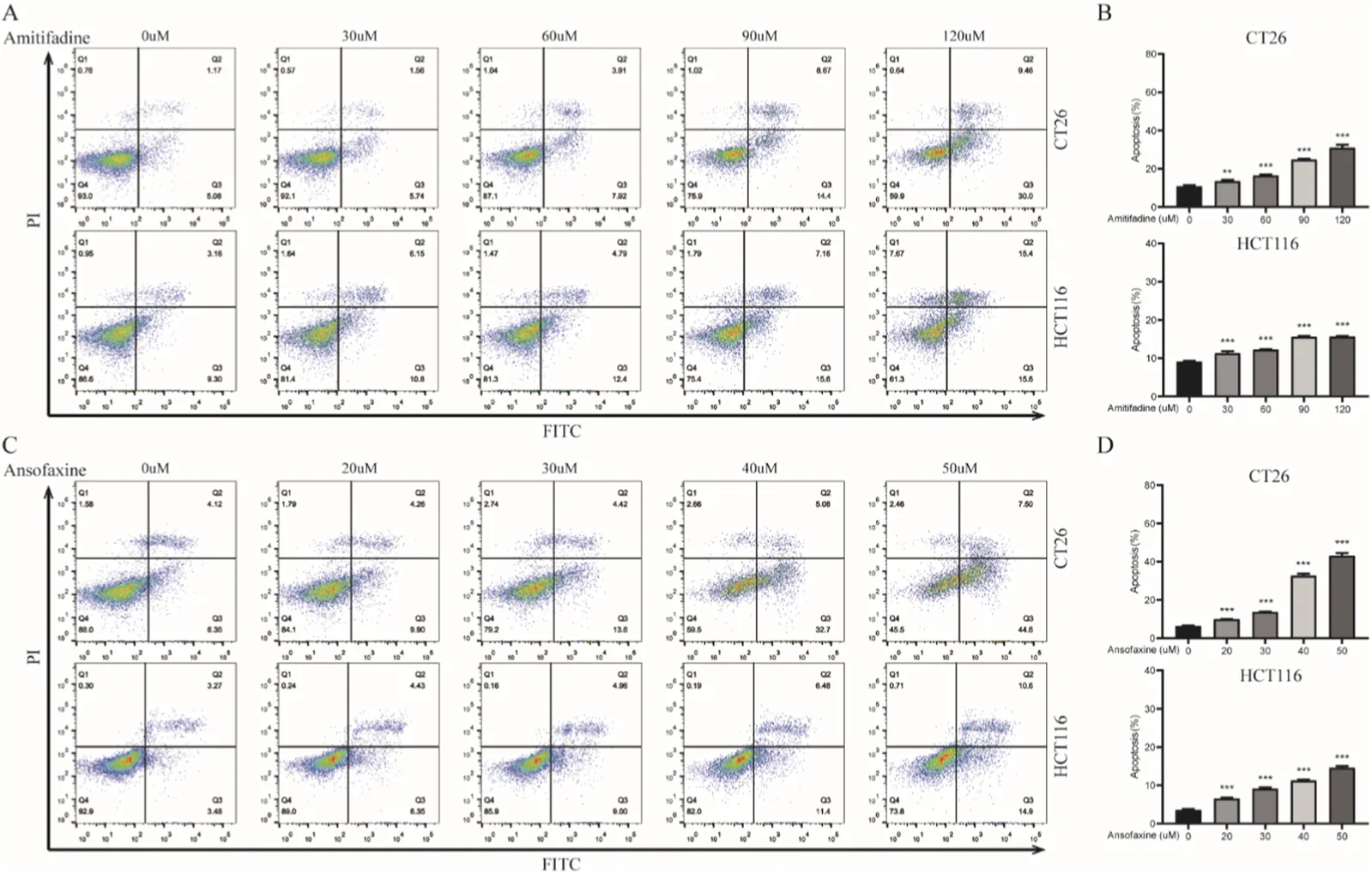
**(A)** CT26 and HCT116 were treated with amitifadine hydrochloride (0–120 μM) for 24 h. **(B)** The proportion of apoptosis cells was analyzed. **(C)** CT26 and HCT116 were treated with ansofaxine hydrochloride (0–50 μM) for 24 h. **(D)** The proportion of apoptosis cells was analyzed.

To investigate the mechanism by which TRIs inhibit tumor cells, we focused on the cell apoptosis. The proportion of apoptotic cells assessed by flow cytometry. Firstly, we found that ansofaxine hydrochloride and amitifadine hydrochloride could induce apoptosis of CT26 and HCT116 cells in a dose-dependent way (Figures 2A, C), with a significant increase in the proportion of apoptotic cells (Figures 2B, D). In addition, the apoptosis of MCA38 and SW620 cell lines was also induced by ansofaxine hydrochloride and amitifadine hydrochloride in a dose-dependent manner (Supplementary Figure S1). On account of the inhibitory effect of ansofaxine hydrochloride on colon cancer cells was stronger than that of amitifadine hydrochloride, we chose ansofaxine hydrochloride for further study.

### 3.3 Ansofaxine hydrochloride inhibited tumor growth

**FIGURE 3:**
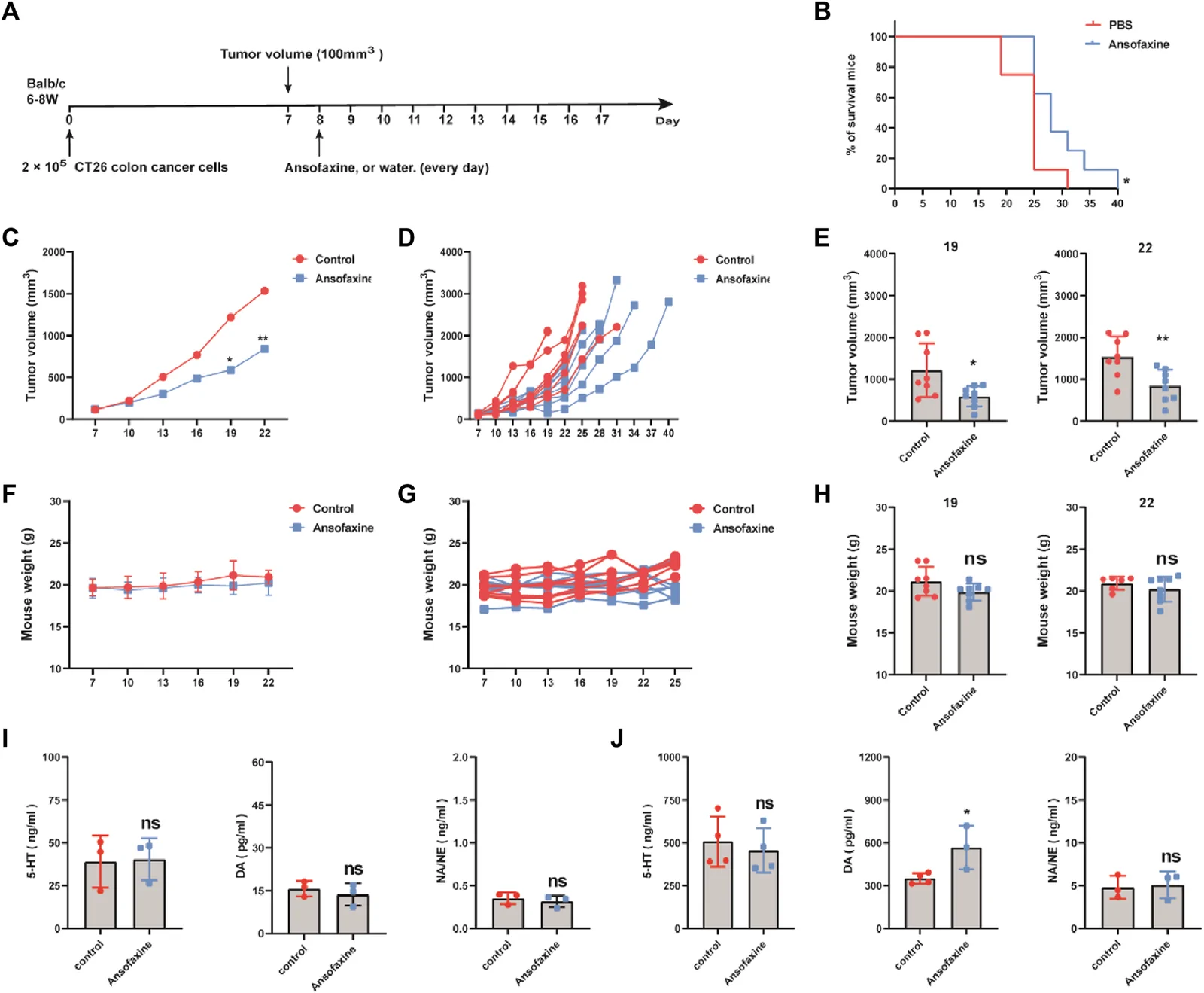
**(A)** Schematic diagram of the experimental program. When tumor volume reached 100 mm^3^ (day 7), mice were then treated with water or ansofaxine hydrochloride for 9 days (300 ug/100 ul/mouse). **(B)** Mouse survival curves. **(C)** Average growth curves of the tumors (*n* = 8). **(D)** Growth curves of tumor in each individual mouse (*n* = 8). **(E)** Tumor volume in mice on days 19 and 22. **(F–H)** Weights of mice. **(I)** Concentrations of 5-HT, DA, and NA/NE in the supernatant of CT26 colon cancer cells treated with or without ansofaxine hydrochloride for 48 h. **(J)** Concentrations of 5-HT, DA, and NA/NE in the serum of mice with and without ansofaxine hydrochloride treatment for 5 days ^ns^ *p* > 0.05, *p < 0.05, **p < 0.01.

Here, we focused on the effects of ansofaxine hydrochloride on the body’s immune system, so we choose mouse CT26 colon cancer cells which are more sensitive to it, are selected for further study. Here, we successfully established CT26 colon cancer mouse model (Figure 3A). Treatment was started when the tumor volume reached 100 mm^3^. Ansofaxine hydrochloride was administered by gavage. Survival analysis showed that ansofaxine hydrochloride improved survival in the CT26 model (Figure 3B). The tumor volume of control group was significantly larger than that of ansofaxine hydrochloride group (Figures 3C–E), there was no change in mouse weight (Figures 3F–H). These results suggest that ansofaxine hydrochloride has anticancer ability *in vivo*.

Next, we investigated the effects of ansofaxine hydrochloride on neurotransmitter secretion. First, we investigated whether CT26 cells secrete neurotransmitters *in vitro*. The results showed that CT26 cells did not secrete dopamine (Below the lower detection line), secreted small amounts of 5-hydroxytryptamine (5-HT) and norepinephrine (NA/NE), which were not affected by treatment with ansofaxine hydrochloride *in vitro* (Figure 3I). We then investigated the effects of ansofaxine hydrochloride on neurotransmitters *in vivo*. After 5 days of ansofaxine hydrochloride treatment, DA was significantly elevated, 5-HT decreased but did not reach a statistical difference, and there was no significant difference in NA/NE in the serum of the mice.

### 3.4 Ansofaxine hydrochloride enhanced the levels of CD8^+^ T cells, inhibited their dysfunction *in vivo*

**FIGURE 4:**
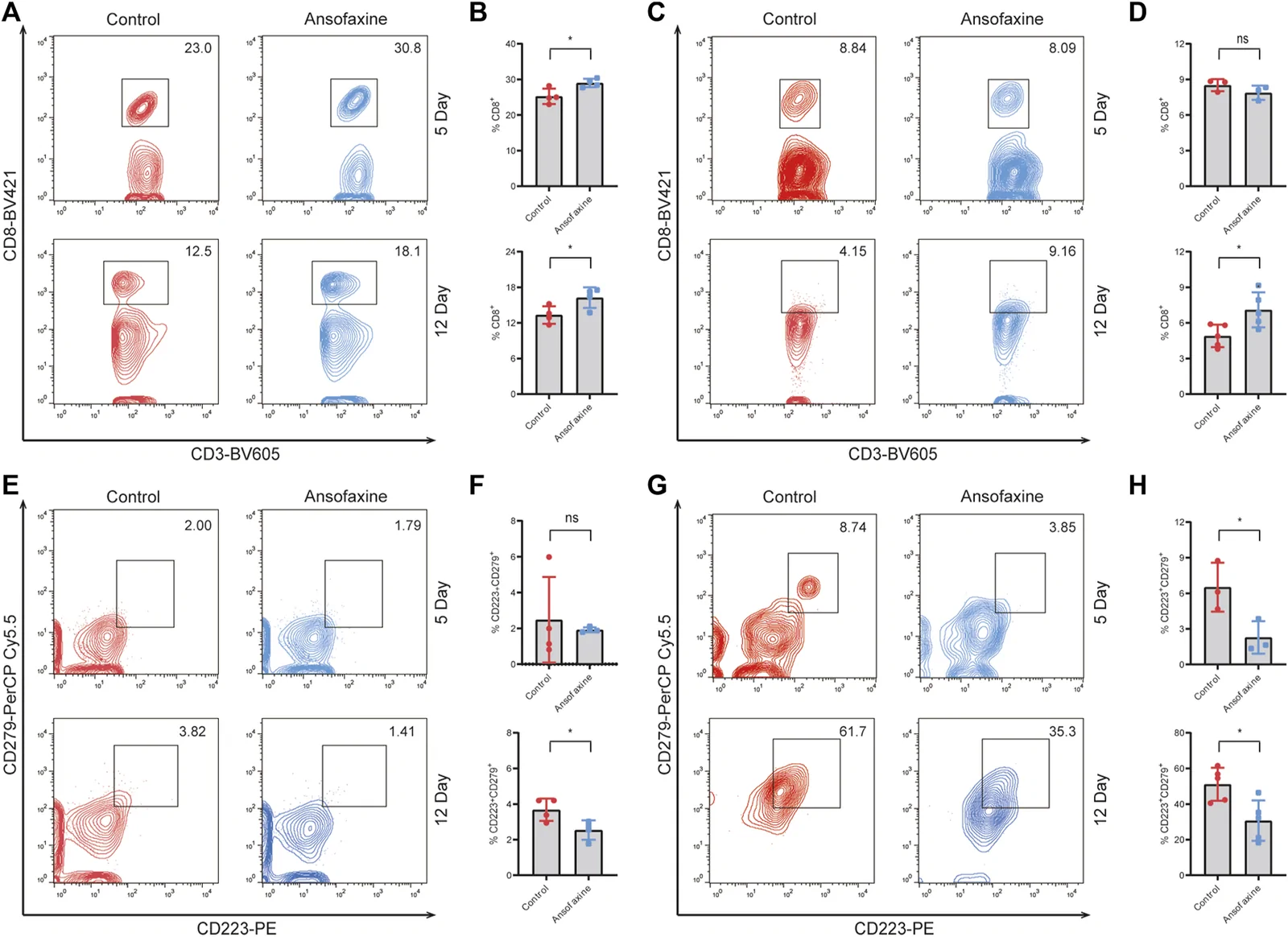
Effects of ansofaxine hydrochloride on CD8^+^ T and exhausted CD8^+^ T *in Vivo*. **(A, B)** The percentages of CD8^+^ in CD3^+^T cells in spleen on days 5 and 12 after ansofaxine hydrochloride administration (*n* = 4). **(C, D)** The percentages of CD8^+^ in CD3^+^T cells in tumor on days 5 (*n* = 3) and 12 (*n* = 4) after ansofaxine hydrochloride administration. **(E, F)** The percentages of CD223^+^CD279^+^ in CD8^+^T cells in spleen on days 5 and 12 after ansofaxine hydrochloride administration (n = 4). **(G, H)** The percentages of CD223^+^CD279^+^ in CD8^+^T cells in tumor on days 5 (*n* = 3) and 12 (*n* = 5) after ansofaxine hydrochloride administration. Data were expressed as mean ± SD. ^ns^ *p* > 0.05, *p < 0.05.

Next, we explored the influence of ansofaxine hydrochloride therapy on the activity and phenotype of T cells. Mice bearing tumors were executed on days 5 and 12 post treatment initiation, and spleens and tumors were extracted for flow cytometry analysis. The results showed a significant increase in the number of CD8^+^ T cells in spleens on days 5 and 12 after ansofaxine hydrochloride administration (Figures 4A, B), while intra-tumoral CD8^+^ T cells no significant change on days 5 after treatment, and significant increased on days 12 after treatment (Figures 4C, D). In addition, the exhausted [programmed death receptor 1 (PD-1)/CD279 and lymphocyte activation gene 3 (LAG3)/CD223] markers showed reduced rates in tumors and spleens in the ansofaxine hydrochloride treatment group (Figures 4E–H).

### 3.5 Ansofaxine hydrochloride enhanced the levels of M1 and NK cells *in vivo*

**FIGURE 5:**
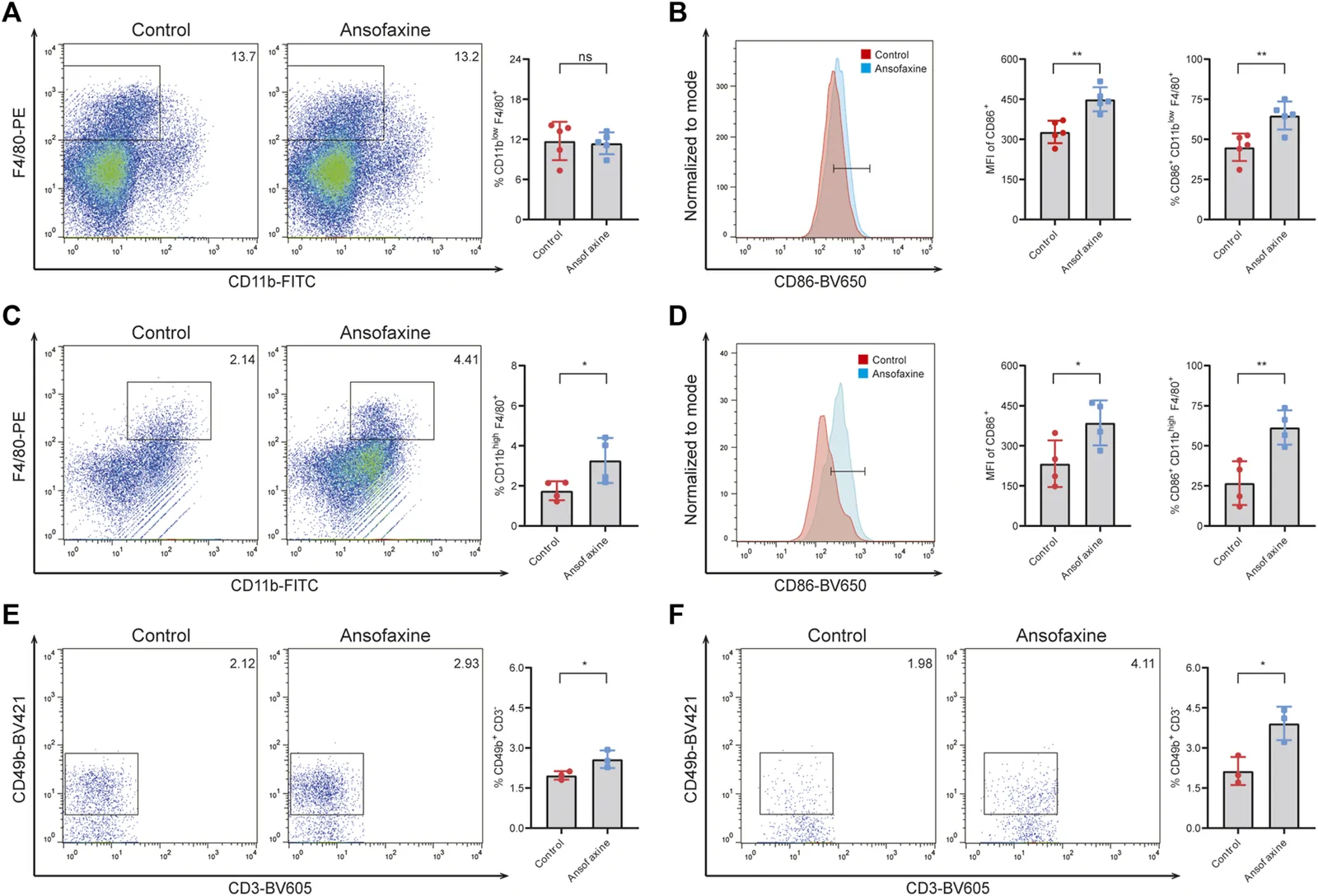
Effects of ansofaxine hydrochloride on M1 and NK cells *in Vivo*. **(A)** The percentages of CD11b^low^ F4/80^+^ M cells in CD45^+^ cells in the spleens on days 5 after ansofaxine hydrochloride administration (*n* = 5). **(B)** The percentages and MFI of CD86^+^ in M cells in the spleens on days 5 after ansofaxine hydrochloride administration (*n* = 5). **(C)** The percentages of CD11b^high^ F4/80^+^ M cells in CD45^+^ cells in the tumor tissues on days 5 after ansofaxine hydrochloride administration (*n* = 4). **(D)** The percentages and MFI of CD86^+^ M1 cells in M cells in the tumor tissues on days 5 after ansofaxine hydrochloride administration (*n* = 4). **(E)** The percentages of CD49b^+^ CD3^−^ NK cells in CD45^+^ cells in spleens on days 5 after ansofaxine hydrochloride administration (*n* = 3). **(F)** The percentages of CD49b^+^ CD3^−^ NK cells in CD45^+^ cells in tumor on days 5 after ansofaxine hydrochloride administration (*n* = 3). Data were presented as mean ± SD. ^ns^ *p* > 0.05, *p < 0.05, **p < 0.01.

Moreover, there was no change in CD11b^low^ F4/80^+^ macrophages in spleen (Figure 5A), but there was a significant increase in M1 (CD11b^low^ F4/80^+^ CD86^+^) (Figure 5B) in spleen on days 5 after ansofaxine hydrochloride treatment. There was significant increase in CD11b^high^ F4/80^+^ macrophages (Figure 5C) and M1 (CD11b^high^ F4/80^+^ CD86^+^) (Figure 5D) in tumor. We also found that the percentages of NK (CD49b^+^ CD3^−^) cells in spleen and tumor significant increased on days 5 after ansofaxine hydrochloride treatment (Figures 5E–F).

### 3.6 Combination therapy with ansofaxine hydrochloride and anti-TNFR2 potently inhibits the growth of CT26 tumors

**FIGURE 6:**
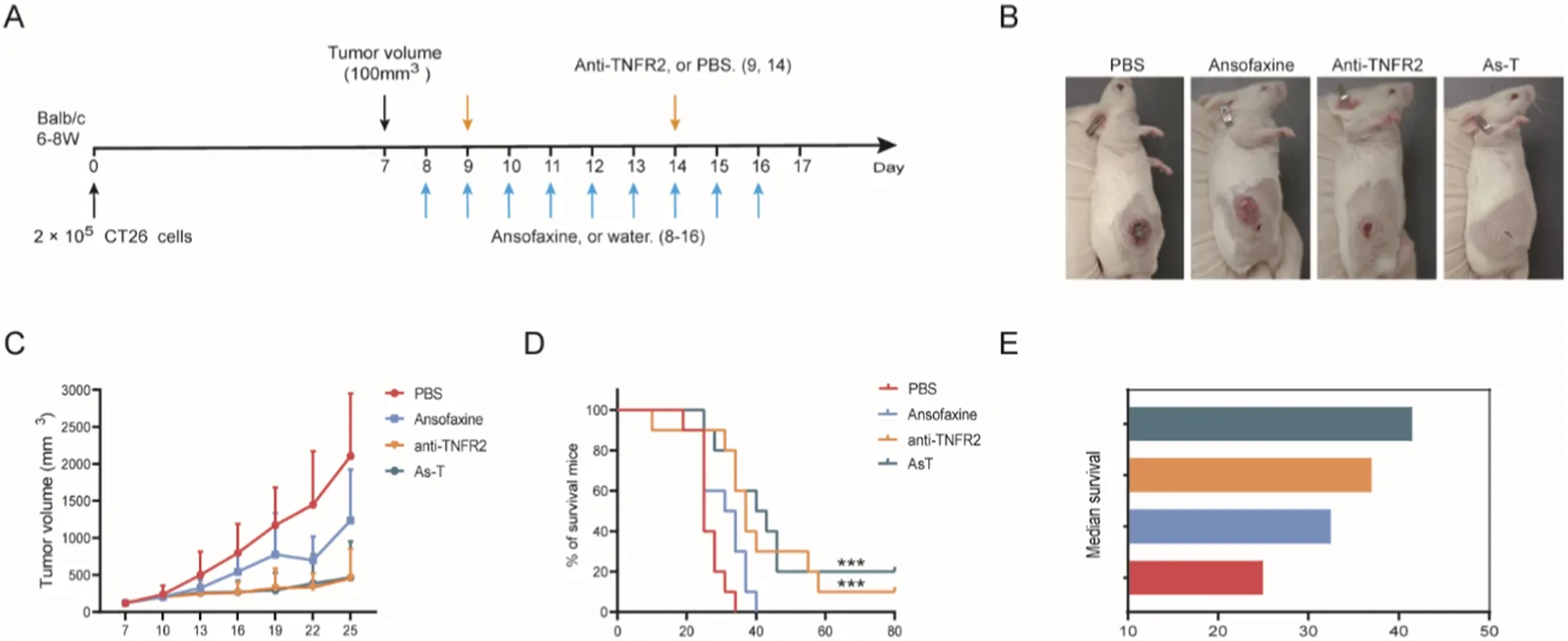
Combination therapy with ansofaxine hydrochloride and anti-TNFR2 potently inhibits the growth of CT26 tumors. **(A)** Schematic diagram of the experimental program. When tumor volume reached 100 mm3 (day 7), mice were then treated with water or ansofaxine hydrochloride (8–16, 300 ug/ 100 ul/mouse), PBS or anti-TNFR2 (9, 14, 200 ug/200 ul/mouse). **(B)** Representative images of mouse on day 19. **(C)** Average growth curves of the tumors (n = 10). **(D)** Mouse survival curves (n = 10). **(E)** Median survival. ns *p* > 0.05, ***p < 0.001.

To investigate the effect of ansofaxine hydrochloride on the efficiency of anti-TNFR2 treatment of colon cancer in mice, we treated tumor-bearing mice with anti-TNFR2 and ansofaxine hydrochloride (Figure 6A). Anti-TNFR2 were administered by intraperitoneal injection. The combination of ansofaxine hydrochloride and anti-TNFR2 (As-T) effectively inhibited the growth of colon tumors (Figures 6B, C), 20% of mice were tumor-free and survived for 80 days, which is the end of the experiment (Figure 6D). Although the tumor growth curve of As-T therapy was not significantly different compared with anti-TNFR2 alone, the median survival of tumor-bearing mice was longer after As-T therapy (41.5 days) compared with anti-TNFR2 (37 days) or ansofaxine hydrochloride (32.5 days) monotherapy (Figure 6E).

### 3.7 Combination therapy with ansofaxine hydrochloride and anti-TNFR2 increases the proportion of CD8^+^ and CD4^+^ T cells in spleen, and reduces the proportion of intra-tumoral Tregs and exhausted CD8^+^ T cells

**FIGURE 7:**
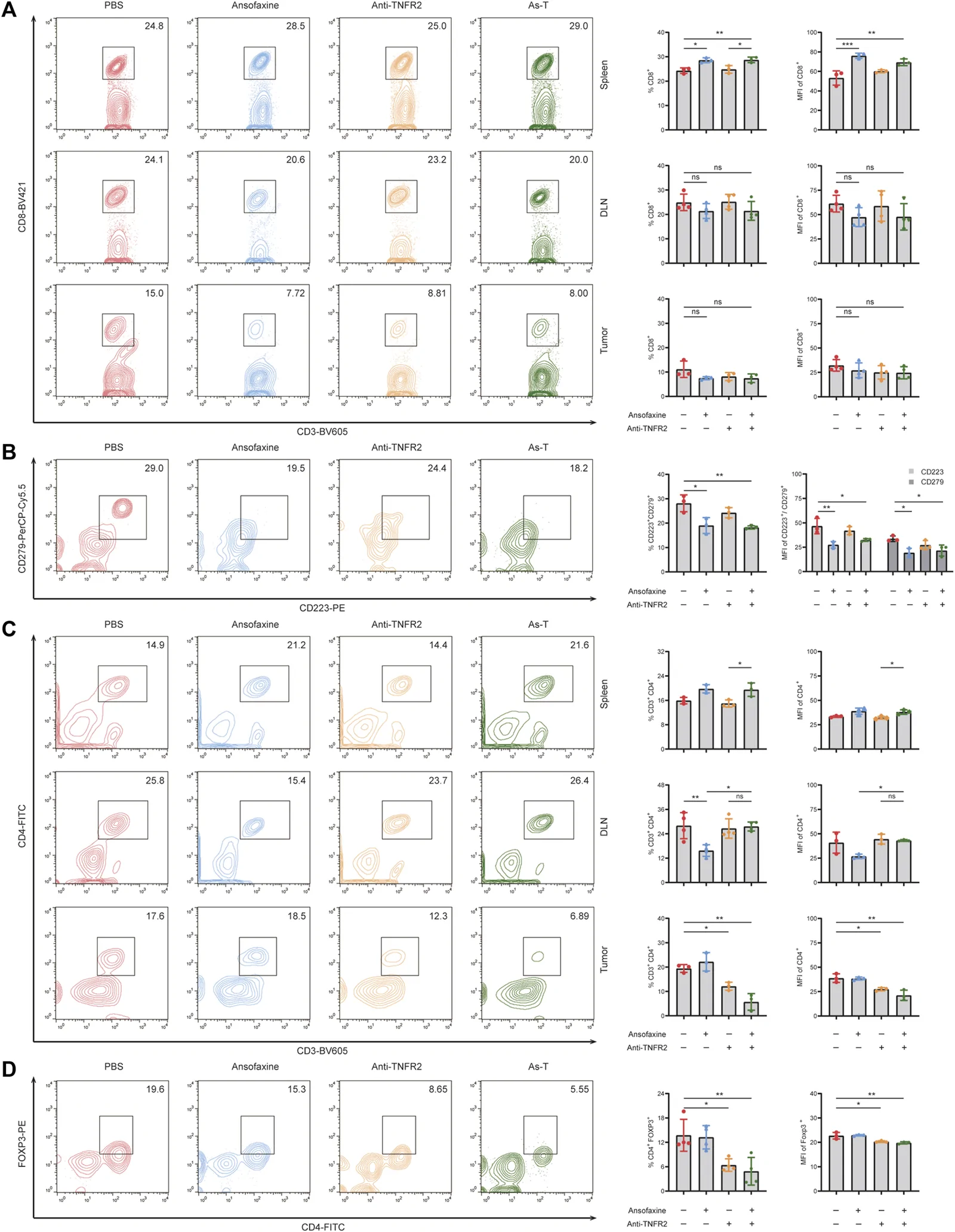
Effects of ansofaxine hydrochloride in combination with anti-TNFR2 on CD8^+^ and CD4^+^ T cells. **(A)** The percentages and MFI of CD8^+^ T cells in CD3^+^ cells in spleen, DLN and tumor (*n* = 3). **(B)** The percentages and MFI of CD223^+^ CD279^+^ cells in CD8^+^ T cells in tumor (*n* = 3). **(C)** The percentages and MFI of CD3^+^CD4^+^ in CD45^+^ cells in spleen, DLN and tumor (*n* = 3). **(D)** The percentages and MFI of Tregs in CD3^+^ cells in tumor (*n* = 3). Data were presented as mean ± SD. ^ns^ *p* > 0.05, *p < 0.05, **p < 0.01.

To investigate the effects of As-T therapy on mice’s systemic immunity and tumor immune microenvironment, we studied the distribution of immune cells in mouse spleens, draining lymph nodes (DLN), and tumors by flow cytometry. The proportion of CD8^+^T and CD4^+^T cells in spleen were significantly increased by treatment with As-T combination therapy, as compared with Anti-TNFR2 alone (Figures 7A, C). However, there was no significant change in DLN. The proportion of tumor-infiltrating CD8 T cells was no significantly change, and tumor-infiltrating CD4^+^T cells was significantly decrease by treatment with As-T combination therapy (Figures 7A, C). Furthermore, As-T combination therapy also significantly reduced the amount of CD223^+^ and CD279^+^ on intra-tumor CD8^+^T cells (Figure 7B), and CD4^+^FOXP3^+^ Treg cells (Figure 7D). Thus, our data suggest that As-T combination therapy enhanced mice’s systemic immunity, and reduced the ratio of intra-tumor exhausted CD8^+^T cells and Treg cells, thereby enhanced anti-tumor immune responses.

### 3.8 Combination therapy with ansofaxine hydrochloride, HMGN1 and 3M-052 potently inhibits the growth of CT26 tumors

**FIGURE 8:**
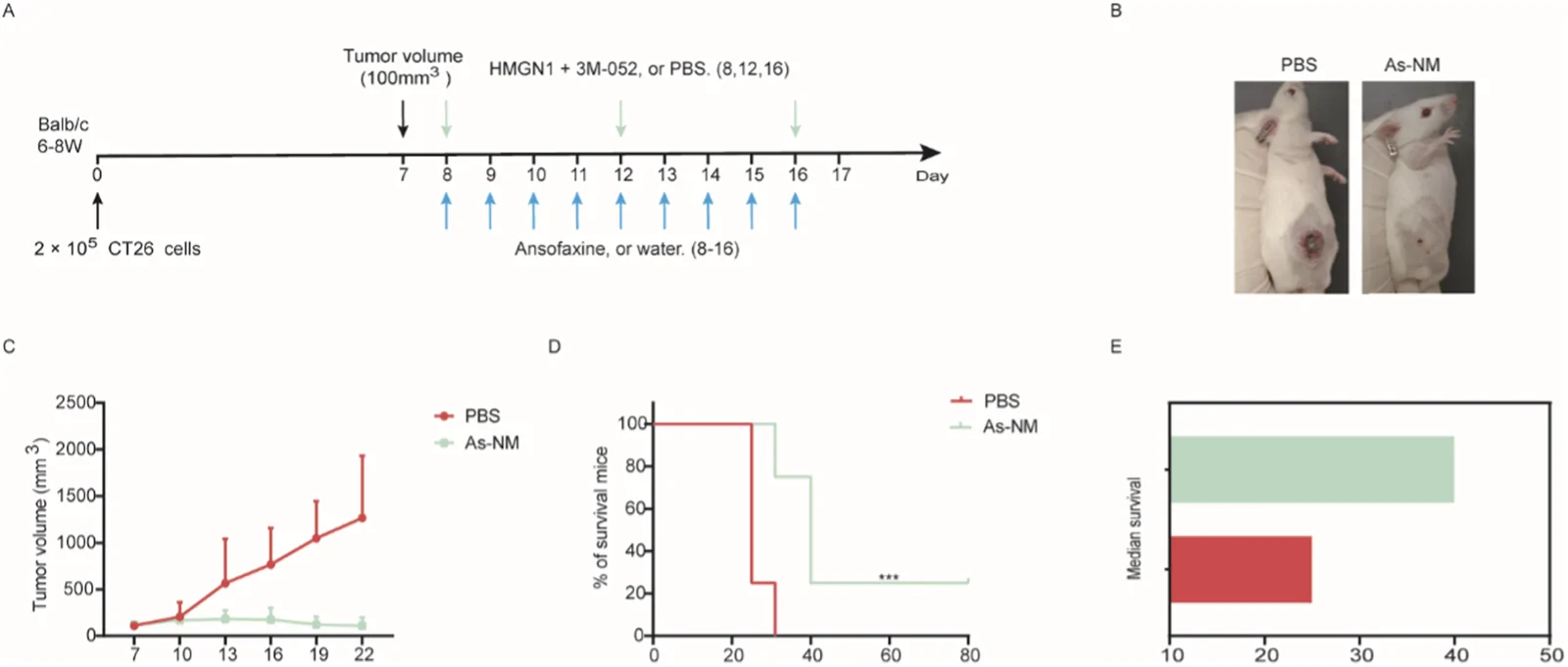
**(A)** Schematic diagram of the experimental program. When tumor volume reached 100 mm^3^ (day 7), mice were then treated with water or ansofaxine hydrochloride (8–16, 300 ug/100 ul/mouse), PBS or HMGN1 (8,12,16, 0.5 ug/50 ul/mouse) and 3M-052 (8,12,16, 20 ug/50 ul/mouse). **(B)** Representative images of mouse on day 19. **(C)** Average growth curves of the tumors (n = 4). **(D)** Mouse survival curves (n = 4). **(E)** Median survival. **p < 0.01.

In addition, we also evaluated the efficacy of ansofaxine hydrochloride combination with immunotherapy targeting dendritic cells. Our previous studies have shown that HMGN1 [toll-like receptor (TLR) 4 agonist] and 3M-052 (TLR7/8 agonist) can work synergistically to stimulate dendritic cell activation (Zhu et al., 2023). HMGN1 and 3M-052 by intratumoral injection (Figure 6A). The combination of ansofaxine hydrochloride and HMGN1 and 3M-052 (As-NM) effectively inhibited the growth of colon tumors (Figures 8B, C), and one mouse was tumor-free and survived for 80 days (Figure 8D). Survival of As-NM treatment was significantly longer than that of control (Figure 8E).

### 3.9 Re-challenge experiment in tumor-free mice

**FIGURE 9:**
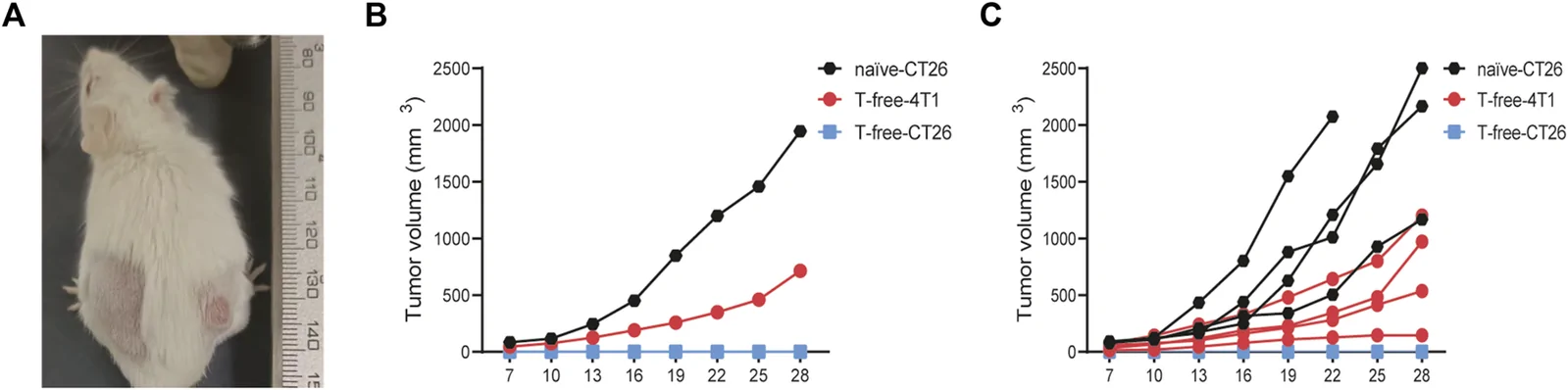
**(A)** Representative images of tumor-free mice rechallenged. **(B, C)** Growth curves of the tumors of tumor-free (T-free) and naïve mice (*n* = 4).

To determine whether the tumor-free mice produced long-term tumor-specific systemic immunity, CT26 cells were reinoculated subcutaneously into the left abdomen of tumor-free mice, and 4T1 breast cancer cells were inoculated into their right abdomen. Following the same method, CT26 cells were injected subcutaneously into naïve mice as control. And unsurprisingly, on day 28 after inoculation, all naïve mice developed CT26 tumors, all tumor-free mice developed 4T1 tumors, but none of these mice developed CT26 tumors (Figure 9B). These data show that the tumor-free mice after treatment produced long-term development of tumor antigen-specific immunity.

## 4 Discussion

In this study, we confirmed that ansofaxine hydrochloride had antitumor effects, possibly by enhancing anti-tumor immune response. Ansofaxine hydrochloride enhanced the effect of immunotherapy with Anti-TNFR2 against CT26 tumors, which was attributable to the reduction in Treg quantity and the recovery of intra-tumor CD8^+^T cells function.

CD8^+^T cells are key effector cells of anti-tumor immunity, however, immunosuppressive and stromal cells in the tumor microenvironment (TME) may regulate intercellular signaling, and the expression levels of cytokines and receptors, ultimately leading to the development of their “exhausted” state (Dolina et al., 2021). Exhausted CD8^+^T cells are lowly responsive to tumor cells and may lead to poor response to ICB therapy, characterized by impaired proliferation and viability, and co-expression of various inhibitory receptors (Wherry and Kurachi, 2015). In general, the greater the number of inhibitory receptors, including lymphocyte activation gene 3 (Lag-3), T-cell immunoglobulin domain and mucin domain protein 3 (Tim-3), CD244, CD160, T-cell immunoreceptor with immunoglobulin and ITIM domains (TIGIT), and cytotoxic T-lymphocyte-associated protein 4 (CTLA-4), co-expressed by exhausted CD8^+^T cells, the more severe the exhaustion. These co-expression patterns are mechanically correlated, and blocking multiple inhibitory receptors simultaneously synergistically inhibits CD8^+^T-cell exhaustion. Some researchers have suggested that peripheral cells may be active reservoirs of exhausted CD8^+^T cells functional precursors prior to physical tumor invasion and chronic exposure to tumor-derived antigens (Dolina et al., 2021). Comparing the tumor with normal neighboring tissue and peripheral blood by scTCR-Seq, it was found that the ICB appeared to primarily mobilize effector CD8^+^T cells from the periphery into the tumor (Wu et al., 2020). In here, we demonstrated that ansofaxine hydrochloride significantly increased the number of peripheral CD8^+^T cells, and reducing the percentages of intra-tumor exhausted CD8^+^T cells. Interestingly, the number of CD8^+^ T cells in the tumor no significantly change after 5 days of ansofaxine hydrochloride treatment, which may be increased consumption of CD8^+^ T cells due to the restoration of anti-tumor effects. Moreover, we also found a significantly increased ratio of M1 macrophages and NKs in spleen and tumor after ansofaxine hydrochloride treatment. In summary, we concluded that ansofaxine hydrochloride remodeled the TME and enhanced the anti-tumor immunity in mice. Studies have reported that a variety of neurotransmitters, such as 5-HT, DA and NA/NE, are involved in the regulation of the immune system (Arreola et al., 2015; Arreola et al., 2016; Eduardo et al., 2019). Dopamine expression was positively correlated with CD8^+^ T-cell infiltration and survival in CRC patients. Dopamine treatment promoted the antitumor activity of CD8^+^ T cells by the dopamine-DRD5 signaling pathway, and inhibited CRC growth in mice (Chen et al., 2022). 5-HT in peripheral blood impairs the function of effector CD8 T cells in tumors, and 5-HT depletion reduces the growth of pancreatic and colorectal tumors and increases intratumoral infiltration of CD8 T cells in wild-type mice (Schneider et al., 2021). In our study, DA was significantly elevated and 5-HT was decreased in peripheral blood of mice after ansofaxine hydrochloride treatment, which may explain the enhancement of antitumor immunity.

Tregs are a subset of CD4^+^T cells with significant immunosuppressive effects, characterized by expression of FOXP3(37). A growing body of research suggests that Tregs are key mediators of the tumor immunosuppressive microenvironment (Takeuchi and Nishikawa, 2016). Tregs secrete immunosuppressive cytokines, inhibit the synthesis and secretion of inflammatory factors, downregulate the expression of major histocompatibility complex class II (MHC II) molecules, and inhibit the expression of co-stimulatory molecules (CD80, CD86) on antigen presenting cells (APC), and finally inhibit T-cell response (Zhang et al., 2021). Removal or inactivation of Tregs in TME is considered as a cancer immunotherapy strategy to activate tumor immunity (Govindaraj et al., 2013; Whiteside, 2018). The most inhibitory Tregs express excess TNFR2, which plays a critical role in the activation and expansion of Tregs, on their surfaces in human cancers (Govindaraj et al., 2013; Govindaraj et al., 2014; Liao et al., 2023). Recent studies have demonstrated that anti-TNFR2, as a potential cancer immunotherapy, produces powerful anti-tumor effects and long-lasting protective memory in a variety of mouse tumor models, and that the mechanism of enhancing anti-tumor immunity may be through the disinhibition of Tregs and exhausted CD8^+^T cells (Torrey et al., 2019; He et al., 2023; Liao et al., 2023). However, other studies have reported that anti-TNFR2 mediates potent co-stimulation of FC-dependent T cells, leading to expansion and improved function of CD8^+^ T cells, without causing significant depletion of Tregs (Tam et al., 2019). In this study, we found that anti-TNFR2 significantly reduced the abundance of Tregs, while the reduction in exhausted CD8^+^T was not statistically different compared with control.

In clinical treatment, the combination of anti-cancer drugs has become routine, and an important consideration is that one treatment may regulate the efficacy of the other. Therefore, we further investigated the animal experimental study of ansofaxine hydrochlorid combined with anti-TNFR2 to confirm whether ansofaxine hydrochlorid has a synergistic anti-tumor effect. Our data showed that ansofaxine hydrochloride not only did not impair the inhibition of Tregs by anti-TNFR2, but also enhanced the reduction of intratumoral exhausted CD8^+^T. In addition, the combination therapy As-T enhanced the number of peripheral CD8^+^T and CD4^+^T cells, compared with anti-TNFR2 therapy alone. This provides new theories and therapeutic amplification for cancer treatment. In addition, we explored the effects of a combination therapy of ansofaxine hydrochlorid and dendritic cell-targeting immunotherapy (As-NM), which resulted in complete tumor elimination in one in four mice, and produced long-term development of tumor antigen-specific immunity. Overall, our work suggests that combined ansofaxine hydrochloride may be a promising approach to cancer treatment.

## 5 Conclusion

In summary, our data reveal the role of ansofaxine hydrochloride in modulating the anti-tumor immunity. Our results support that exhausted CD8^+^T is an important potential mechanism by which ansofaxine hydrochloride activates anti-tumor immunity and enhances anti-tumor effects of anti-TNFR2. In addition, the study presented here suggests that strategic combination with ansofaxine hydrochloride may enhance the efficacy of tumor immunotherapy in colon cancer.

## Data Availability

The original contributions presented in the study are included in the article/Supplementary Material, further inquiries can be directed to the corresponding author.

## References

[B1] Arreola R. Alvarez-Herrera S. Pérez-Sánchez G. Becerril-Villanueva E. Cruz-Fuentes C. Flores-Gutierrez E. O. (2016). Immunomodulatory effects mediated by dopamine. J. Immunol. Res. 2016, 3160486–3160531. 10.1155/2016/3160486 27795960 PMC5067323

[B2] Arreola R. Becerril-Villanueva E. Cruz-Fuentes C. Velasco-Velázquez M. A. Garcés-Alvarez M. E. Hurtado-Alvarado G. (2015). Immunomodulatory effects mediated by serotonin. J. Immunol. Res. 2015, 1–21. 10.1155/2015/354957 25961058 PMC4417587

[B3] Chen X. Bäumel M. Männel D. N. Howard O. M. Z. Oppenheim J. J. (2007). Interaction of TNF with TNF receptor type 2 promotes expansion and function of mouse CD4+CD25+ T regulatory cells. J. Immunol. 179 (1), 154–161. 10.4049/jimmunol.179.1.154 17579033

[B4] Chen Y. Yan S.-M. Pu Z. Feng J. Tan L. Li Y. (2022). Dopamine signaling promotes tissue-resident memory differentiation of CD8+ T cells and antitumor immunity. Cancer Res. 82 (17), 3130–3142. 10.1158/0008-5472.CAN-21-4084 35802647

[B5] Di Rosso M. E. Palumbo M. L. Genaro A. M. (2016). Immunomodulatory effects of fluoxetine: a new potential pharmacological action for a classic antidepressant drug? Pharmacol. Res. 109, 101–107. 10.1016/j.phrs.2015.11.021 26644208

[B6] Di Rosso M. E. Sterle H. A. Cremaschi G. A. Genaro A. M. (2018). Beneficial effect of fluoxetine and sertraline on chronic stress-induced tumor growth and cell dissemination in a mouse model of lymphoma: crucial role of antitumor immunity. Front. Immunol. 9, 1341. 10.3389/fimmu.2018.01341 29971064 PMC6018164

[B7] Dolina J. S. Van Braeckel-Budimir N. Thomas G. D. Salek-Ardakani S. (2021). CD8+ T cell exhaustion in cancer. Front. Immunol. 12, 715234. 10.3389/fimmu.2021.715234 34354714 PMC8330547

[B8] Eduardo C.-R. C. Alejandra T.-I. G. Guadalupe D.-R. K. J. Herminia V.-R. G. Lenin P. Enrique B.-V. (2019). Modulation of the extraneuronal cholinergic system on main innate response leukocytes. J. Neuroimmunol. 327, 22–35. 10.1016/j.jneuroim.2019.01.008 30683425

[B9] Endo M. Matsui K. Akaho R. Mitsui K. Yan Y. Imai Y. (2022). Depressive and anxiety symptoms among Japanese cancer survivors: Japan cancer survivorship research project. BMC Cancer 22 (1), 134. 10.1186/s12885-022-09215-x 35109805 PMC8811965

[B10] Fritz J. M. Lenardo M. J. (2019). Development of immune checkpoint therapy for cancer. J. Exp. Med. 216 (6), 1244–1254. 10.1084/jem.20182395 31068379 PMC6547853

[B11] Govindaraj C. Scalzo-Inguanti K. Madondo M. Hallo J. Flanagan K. Quinn M. (2013). Impaired Th1 immunity in ovarian cancer patients is mediated by TNFR2+ Tregs within the tumor microenvironment. Clin. Immunol. 149 (1), 97–110. 10.1016/j.clim.2013.07.003 23948613

[B12] Govindaraj C. Tan P. Walker P. Wei A. Spencer A. Plebanski M. (2014). Reducing TNF receptor 2+ regulatory T cells via the combined action of azacitidine and the HDAC inhibitor, panobinostat for clinical benefit in acute myeloid leukemia patients. Clin. Cancer Res. 20 (3), 724–735. 10.1158/1078-0432.CCR-13-1576 24297862

[B13] He T. Chen Y. Yang D. Islam M. S. Chou C.-K. Liu J. (2023). TNFR2 antagonistic antibody induces the death of tumor infiltrating CD4+Foxp3+ regulatory T cells. Cell Oncol. (Dordr). 46 (1), 167–177. 10.1007/s13402-022-00742-0 36369606 PMC12974723

[B14] Kawano S. Mitoma H. Inokuchi S. Yamauchi Y. Yokoyama K. Nogami J. (2022). TNFR2 signaling enhances suppressive abilities of human circulating T follicular regulatory cells. J. Immunol. 208 (5), 1057–1065. 10.4049/jimmunol.2100323 35149531

[B15] Lee H.-C. Chiu W.-C. Wang T.-N. Liao Y.-T. Chien I. C. Lee Y. (2017). Antidepressants and colorectal cancer: a population-based nested case-control study. J. Affect. Disord. 207, 353–358. 10.1016/j.jad.2016.09.057 27744223

[B16] Li P. Yang Y. Yang X. Wang Y. Chou C.-K. Jiang M. (2023). TNFR2 deficiency impairs the growth of mouse colon cancer. Int. J. Biol. Sci. 19 (4), 1024–1035. 10.7150/ijbs.72606 36923938 PMC10008691

[B17] Liao P. Jiang M. Islam M. S. Wang Y. Chen X. (2023). TNFR2 expression predicts the responses to immune checkpoint inhibitor treatments. Front. Immunol. 14, 1097090. 10.3389/fimmu.2023.1097090 36865537 PMC9971721

[B18] Liu J. Deng G.-H. Zhang J. Wang Y. Xia X.-Y. Luo X.-M. (2015). The effect of chronic stress on anti-angiogenesis of sunitinib in colorectal cancer models. Psychoneuroendocrinology 52, 130–142. 10.1016/j.psyneuen.2014.11.008 25437118

[B19] Marcinkute M. Afshinjavid S. Fatokun A. A. Javid F. A. (2019). Fluoxetine selectively induces p53-independent apoptosis in human colorectal cancer cells. Eur. J. Pharmacol. 857, 172441. 10.1016/j.ejphar.2019.172441 31181210

[B20] Mi W. Yang F. Li H. Xu X. Li L. Tan Q. (2022). Efficacy, safety, and tolerability of ansofaxine (LY03005) extended-release tablet for major depressive disorder: a randomized, double-blind, placebo-controlled, dose-finding, phase 2 clinical trial. Int. J. Neuropsychopharmacol. 25 (3), 252–260. 10.1093/ijnp/pyab074 34747448 PMC8929756

[B21] Moatti A. Debesset A. Pilon C. Beldi-Ferchiou A. Leclerc M. Redjoul R. (2022). TNFR2 blockade of regulatory T cells unleashes an antitumor immune response after hematopoietic stem-cell transplantation. J. Immunother. Cancer 10 (4), e003508. 10.1136/jitc-2021-003508 35387779 PMC8987798

[B22] Schneider M. A. Heeb L. Beffinger M. M. Pantelyushin S. Linecker M. Roth L. (2021). Attenuation of peripheral serotonin inhibits tumor growth and enhances immune checkpoint blockade therapy in murine tumor models. Sci. Transl. Med. 13 (611), eabc8188. 10.1126/scitranslmed.abc8188 34524861

[B23] Shao L. Li W. Xie Q. Yin H. (2014). Triple reuptake inhibitors: a patent review (2006 - 2012). Expert Opin. Ther. Pat. 24 (2), 131–154. 10.1517/13543776.2014.859676 24289044

[B24] Sharma H. Santra S. Dutta A. (2015). Triple reuptake inhibitors as potential next-generation antidepressants: a new hope? Future Med. Chem. 7 (17), 2385–2406. 10.4155/fmc.15.134 26619226 PMC4976848

[B25] Sommershof A. Scheuermann L. Koerner J. Groettrup M. (2017). Chronic stress suppresses anti-tumor TCD8+ responses and tumor regression following cancer immunotherapy in a mouse model of melanoma. Brain Behav. Immun. 65, 140–149. 10.1016/j.bbi.2017.04.021 28457810

[B26] Sung H. Ferlay J. Siegel R. L. Laversanne M. Soerjomataram I. Jemal A. (2021). Global cancer statistics 2020: GLOBOCAN estimates of incidence and mortality worldwide for 36 cancers in 185 countries. CA Cancer J. Clin. 71 (3), 209–249. 10.3322/caac.21660 33538338

[B27] Takeuchi Y. Nishikawa H. (2016). Roles of regulatory T cells in cancer immunity. Int. Immunol. 28 (8), 401–409. 10.1093/intimm/dxw025 27160722 PMC4986235

[B28] Tam E. M. Fulton R. B. Sampson J. F. Muda M. Camblin A. Richards J. (2019). Antibody-mediated targeting of TNFR2 activates CD8+ T cells in mice and promotes antitumor immunity. Sci. Transl. Med. 11 (512), eaax0720. 10.1126/scitranslmed.aax0720 31578241

[B29] Torrey H. Khodadoust M. Tran L. Baum D. Defusco A. Kim Y. H. (2019). Targeted killing of TNFR2-expressing tumor cells and Tregs by TNFR2 antagonistic antibodies in advanced Sézary syndrome. Leukemia 33 (5), 1206–1218. 10.1038/s41375-018-0292-9 30356161 PMC6756055

[B30] Tran P. Skolnick P. Czobor P. Huang N. Y. Bradshaw M. McKinney A. (2012). Efficacy and tolerability of the novel triple reuptake inhibitor amitifadine in the treatment of patients with major depressive disorder: a randomized, double-blind, placebo-controlled trial. J. Psychiatr. Res. 46 (1), 64–71. 10.1016/j.jpsychires.2011.09.003 21925682

[B31] Vanamee É. S. Faustman D. L. (2017). TNFR2: a novel target for cancer immunotherapy. Trends Mol. Med. 23 (11), 1037–1046. 10.1016/j.molmed.2017.09.007 29032004

[B32] Wang X. Li B. Kim Y. J. Wang Y.-C. Li Z. Yu J. (2021a). Targeting monoamine oxidase A for T cell-based cancer immunotherapy. Sci. Immunol. 6 (59), eabh2383. 10.1126/sciimmunol.abh2383 33990379

[B33] Wang Y.-C. Wang X. Yu J. Ma F. Li Z. Zhou Y. (2021b). Targeting monoamine oxidase A-regulated tumor-associated macrophage polarization for cancer immunotherapy. Nat. Commun. 12 (1), 3530. 10.1038/s41467-021-23164-2 34112755 PMC8192781

[B34] Wherry E. J. Kurachi M. (2015). Molecular and cellular insights into T cell exhaustion. Nat. Rev. Immunol. 15 (8), 486–499. 10.1038/nri3862 26205583 PMC4889009

[B35] Whiteside T. L. (2018). FOXP3+ Treg as a therapeutic target for promoting anti-tumor immunity. Expert Opin. Ther. Targets 22 (4), 353–363. 10.1080/14728222.2018.1451514 29532697 PMC6126897

[B36] Wu T. D. Madireddi S. de Almeida P. E. Banchereau R. Chen Y.-J. J. Chitre A. S. (2020). Peripheral T cell expansion predicts tumour infiltration and clinical response. Nature 579 (7798), 274–278. 10.1038/s41586-020-2056-8 32103181

[B37] Yan R. Xia J. Yang R. Lv B. Wu P. Chen W. (2019). Association between anxiety, depression, and comorbid chronic diseases among cancer survivors. Psycho-oncology 28 (6), 1269–1277. 10.1002/pon.5078 30946503

[B38] Yang W. Xiao L. Yuan Z. Huang H. Xiang Y. Liu Z. (2021a). Anxiety and depression in patients with physical diseases and associated factors: a large-scale field survey in general hospitals in China. Front. Psychiatry 12, 689787. 10.3389/fpsyt.2021.689787 34393853 PMC8359676

[B39] Yang Z. Li Z. Guo Z. Ren Y. Zhou T. Xiao Z. (2021b). Antitumor effect of fluoxetine on chronic stress-promoted lung cancer growth via suppressing kynurenine pathway and enhancing cellular immunity. Front. Pharmacol. 12, 685898. 10.3389/fphar.2021.685898 34413774 PMC8369900

[B40] Zhang L. Pan J. Chen W. Jiang J. Huang J. (2020). Chronic stress-induced immune dysregulation in cancer: implications for initiation, progression, metastasis, and treatment. Am. J. Cancer Res. 10 (5), 1294–1307. 32509380 PMC7269780

[B41] Zhang Y. Guo J. Jia R. (2021). Treg: a promising immunotherapeutic target in oral diseases. Front. Immunol. 12, 667862. 10.3389/fimmu.2021.667862 34177907 PMC8222692

[B42] Zhu L. Zhang X. Chen X. Yang D. Nie Y. Pan R. (2023). Anti-TNFR2 enhanced the antitumor activity of a new HMGN1/3M-052 stimulated dendritic cell vaccine in a mouse model of colon cancer. Biochem. Biophys. Res. Commun. 653, 106–114. 10.1016/j.bbrc.2023.02.039 36868074

