# Correction: Ansofaxine hydrochloride inhibits tumor growth and enhances anti-TNFR2 in murine colon cancer model

**DOI:** 10.3389/fphar.2026.1948074

**Published:** 2026-09-02

**Authors:** Qianyu Jing, Quan Wan, Yujie Nie, Junqian Luo, Xiangyan Zhang, Lan Zhu, Huan Gui, Linzhao Li, Chenglv Wang, Shuanghui Chen, Mengjiao Wang, Haohua Yuan, Hang Lv, Runsang Pan, Qianjun Jing, Yingjie Nie

**Affiliations:** 1 School of Basic Medical Sciences, Zunyi Medical University, Zunyi, China; 2 NHC Key Laboratory of Pulmonary Immunological Diseases, Guizhou Provincial People’s Hospital, Guiyang, China; 3 The First People’s Hospital of Jinzhong, Jinzhong, China; 4 School of Medicine, Guizhou University, Guiyang, China; 5 Guizhou Medical University, Guiyang, China; 6 Chongqing Medical University, Chongqing, China

**Keywords:** ansofaxine hydrochloride, anti-TNFR2, exhausted CD8 + T cells, Tregs, tumor immunotherapy, colon cancer

There was a mistake in Figures 2B,D as published. The method for measuring apoptosis is incorrect. The corrected Figure 2 appears below.

**FIGURE 2:**
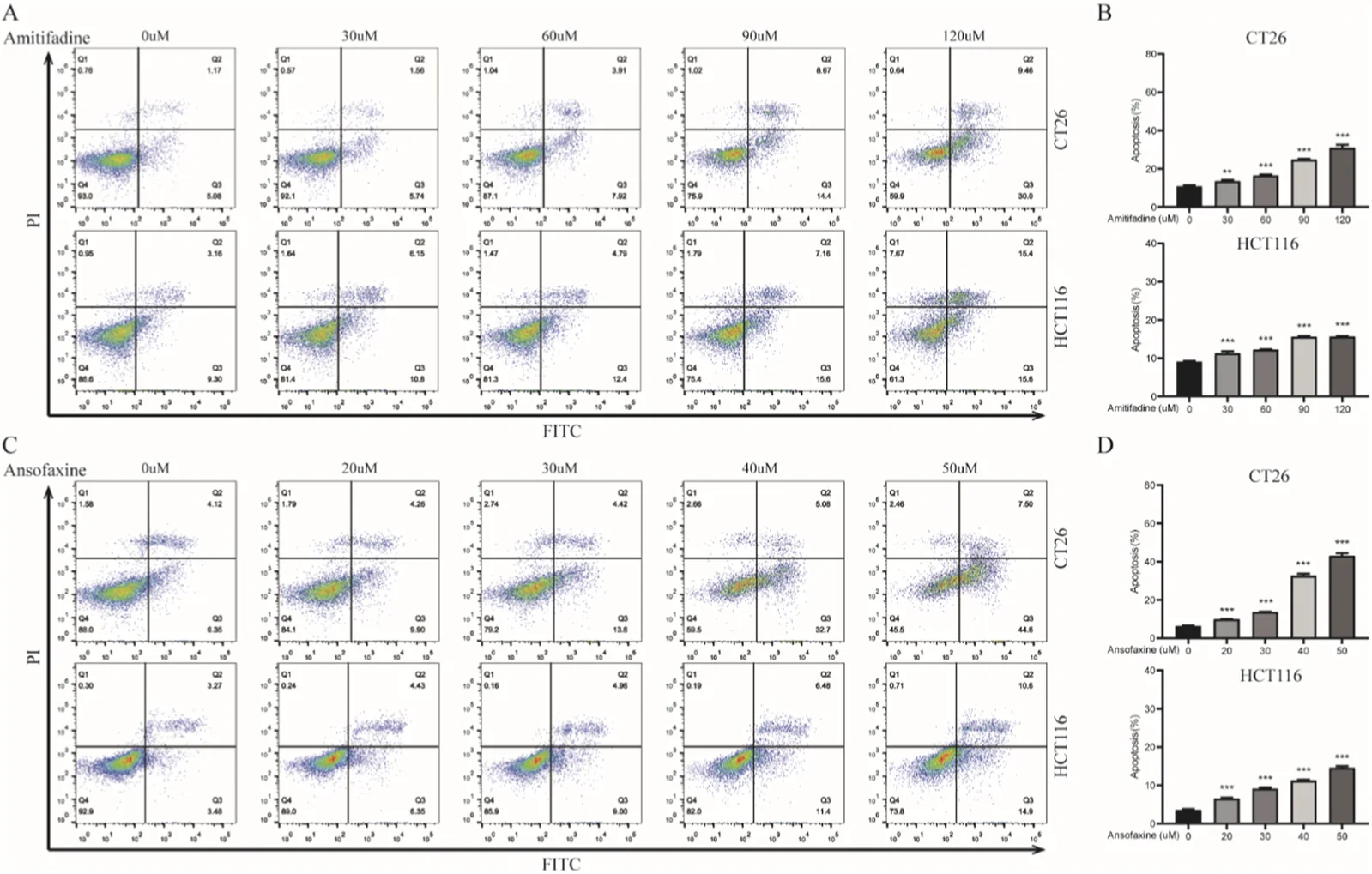
**(A)** CT26 and HCT116 were treated with amitifadine hydrochloride (0–120 μM) for 24 h. **(B)** The proportion of apoptosis cells was analyzed. **(C)** CT26 and HCT116 were treated with ansofaxine hydrochloride (0–50 μM) for 24 h. **(D)** The proportion of apoptosis cells was analyzed.

There was a mistake in Figures 6C, 8C as published. Because the observation period in this study was relatively long, the authors listed the final tumor volumes at the time of death for all mice in Figures 6C, 8C. Recognizing that this could be misleading, the authors have corrected Figures 6C, 8C to show only short-term tumor growth. The corrected Figures 6, 8 appears below.

**FIGURE 6:**
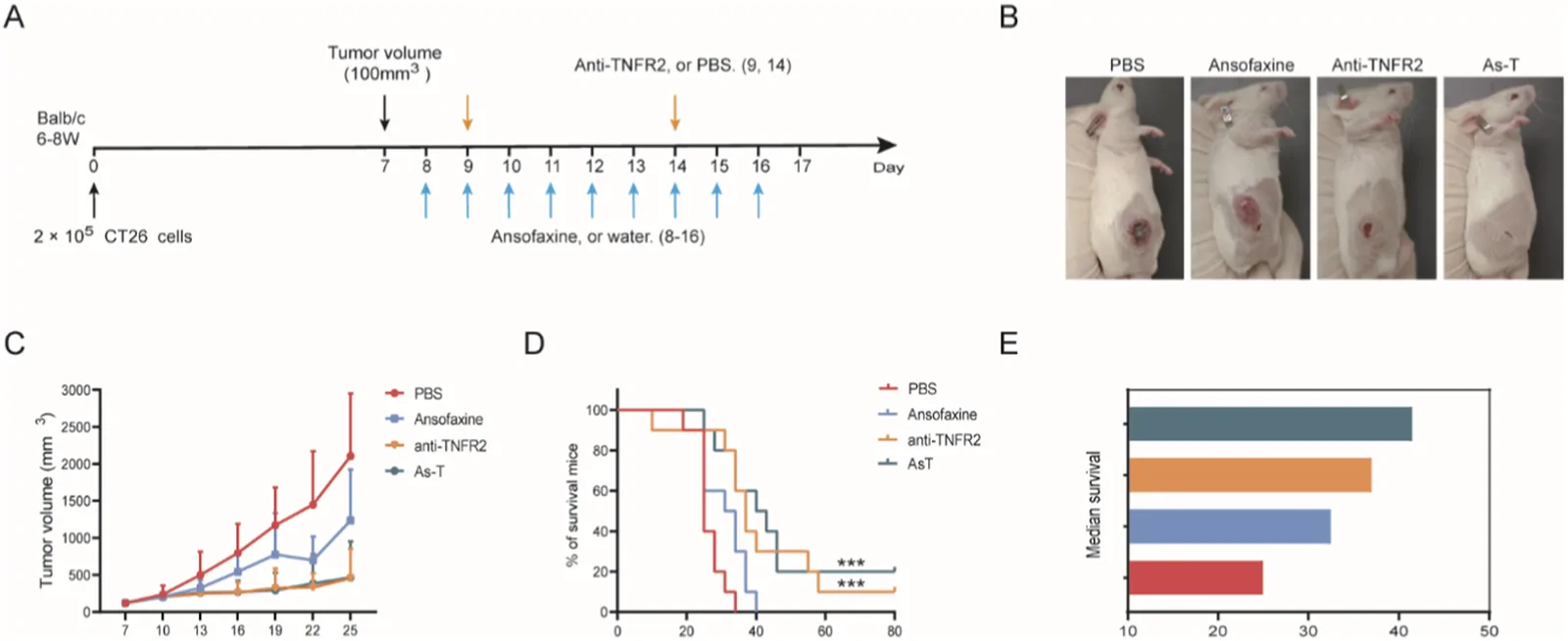
Combination therapy with ansofaxine hydrochloride and anti-TNFR2 potently inhibits the growth of CT26 tumors. **(A)** Schematic diagram of the experimental program. When tumor volume reached 100 mm^3^ (day 7), mice were then treated with water or ansofaxine hydrochloride (8–16, 300 ug/100 ul/mouse), PBS or anti-TNFR2 (9,14, 200 ug/200 ul/mouse). **(B)** Representative images of mouse on day 19. **(C)** Average growth curves of the tumors (n = 10). **(D)** Mouse survival curves (n = 10). **(E)** Median survival. ns p > 0.05, ***p < 0.001.

**FIGURE 8:**
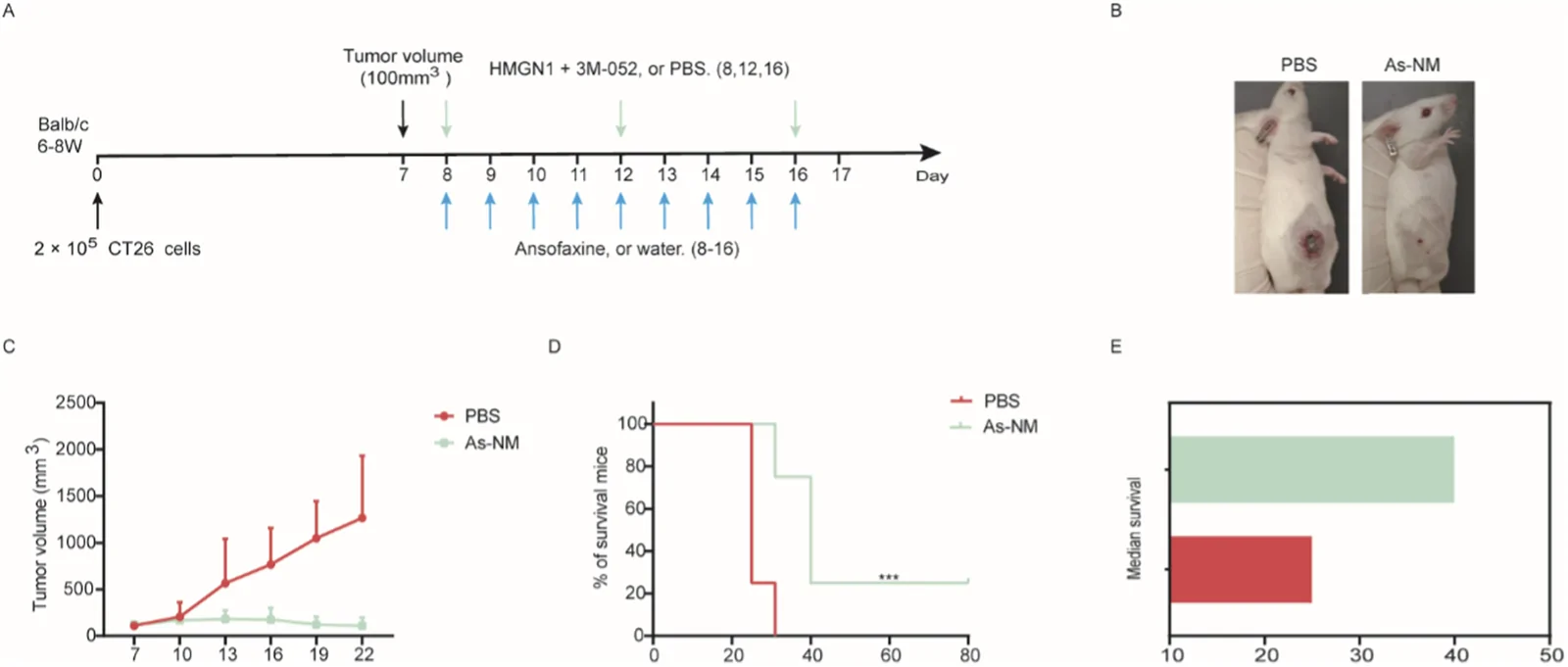
**(A)** Schematic diagram of the experimental program. When tumor volume reached 100 mm^3^ (day 7), mice were then treated with water or ansofaxine hydrochloride (8–16, 300 ug/100 ul/mouse), PBS or HMGN1 (8,12,16, 0.5 ug/50 ul/mouse) and 3M-052 (8,12,16, 20 ug/50 ul/mouse). **(B)** Representative images of mouse on day 19. **(C)** Average growth curves of the tumors (n = 4). **(D)** Mouse survival curves (n = 4). **(E)** Median survival. **p < 0.01.

The original version of this article has been updated.

