# Supplementary material for: Ansofaxine hydrochloride inhibits tumor growth and enhances Anti-TNFR2 in murine colon cancer model
Source: Front Pharmacol. 2023 Dec 14;14:1286061. doi: 10.3389/fphar.2023.1286061 (PMC10755865; doi:10.3389/fphar.2023.1286061)
Supplement: Supplementary file 1 [file DataSheet1.docx]

Ansofaxine hydrochloride inhibits tumor growth and enhances Anti-TNFR2 in murine colon cancer model

Qianyu Jing^a^*, Quan Wan^a^*, Yujie Nie^b^, Junqian Luo^c^, Xiangyan Zhang^b^, Lan Zhu^d^, Huan Gui^d^, Linzhao Li^d^, Chenglv Wang^d^, Shuanghui Chen^d^, Mengjiao Wang^d^, Haohua Yuan^d^, Hang Lv^d^, Runsang Pan^e^, Yingjie Nie^a,^ ^b#^


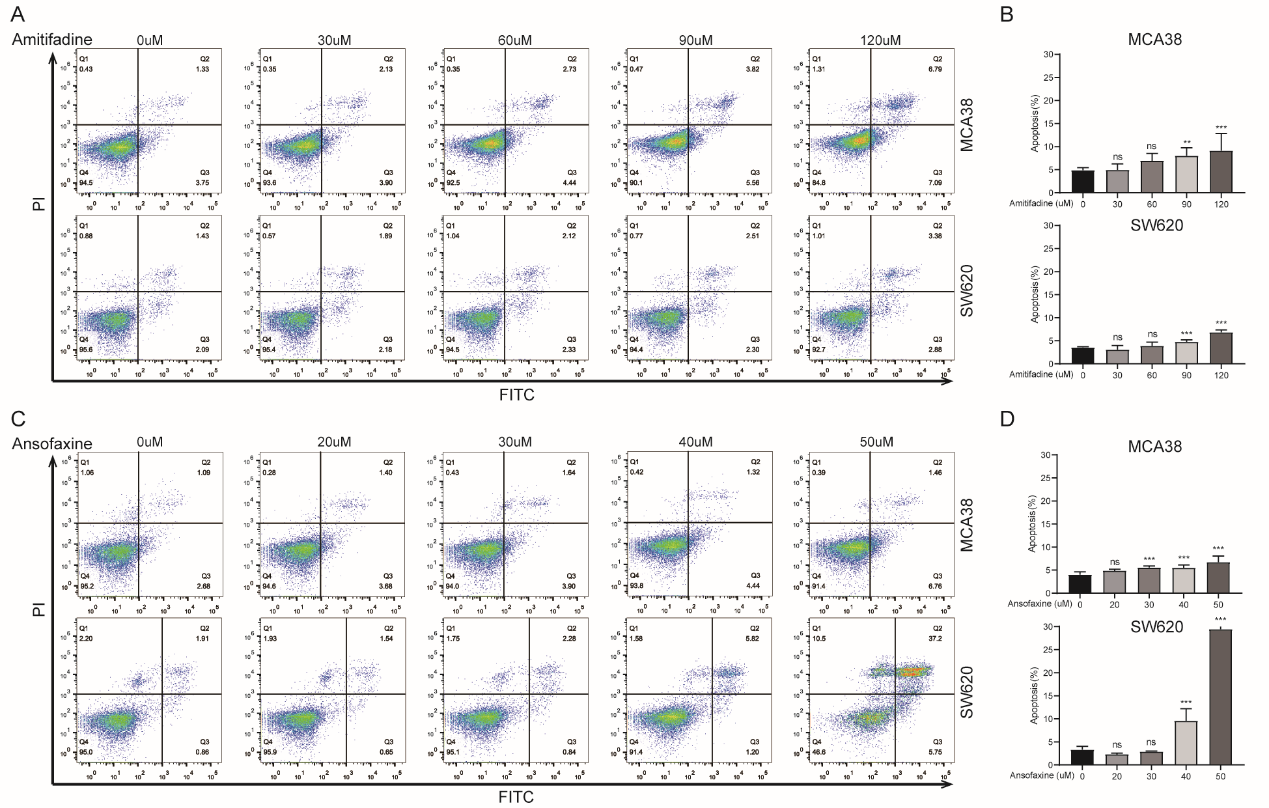


Supplementary Figure S1 **(A)** Cells were treated with Amitifadine hydrochloride (0–120 μM) for 24 h, performed PI/FITC staining, and analyzed with flow cytometry. **(B)** Percentage of apoptosis cells was analyzed. **(C)** Cells were treated with Ansofaxine hydrochloride (0–50 μM) for 24 h, performed PI/FITC staining, and analyzed with flow cytometry. **(D)** Percentage of apoptosis cells was analyzed.
